# Model-based learning protects against forming habits

**DOI:** 10.3758/s13415-015-0347-6

**Published:** 2015-03-24

**Authors:** Claire M. Gillan, A. Ross Otto, Elizabeth A. Phelps, Nathaniel D. Daw

**Affiliations:** 1 Department of Psychology, New York University, 6 Washington Place, New York, NY 10003 USA; 2 Department of Psychology and Behavioural and Clinical Neuroscience Institute, University of Cambridge, Cambridge, UK; 3 Center for Neural Science, New York University, New York, NY USA; 4 Nathan Kline Institute, Orangeburg, NY USA

**Keywords:** Reinforcement learning, Devaluation, Habit, Goal-directed, Model-based, Model-free

## Abstract

Studies in humans and rodents have suggested that behavior can at times be “goal-directed”—that is, planned, and purposeful—and at times “habitual”—that is, inflexible and automatically evoked by stimuli. This distinction is central to conceptions of pathological compulsion, as in drug abuse and obsessive-compulsive disorder. Evidence for the distinction has primarily come from outcome devaluation studies, in which the sensitivity of a previously learned behavior to motivational change is used to assay the dominance of habits versus goal-directed actions. However, little is known about how habits and goal-directed control arise. Specifically, in the present study we sought to reveal the trial-by-trial dynamics of instrumental learning that would promote, and protect against, developing habits. In two complementary experiments with independent samples, participants completed a sequential decision task that dissociated two computational-learning mechanisms, model-based and model-free. We then tested for habits by devaluing one of the rewards that had reinforced behavior. In each case, we found that individual differences in model-based learning predicted the participants’ subsequent sensitivity to outcome devaluation, suggesting that an associative mechanism underlies a bias toward habit formation in healthy individuals.

**Electronic supplementary material:**

The online version of this article (doi:10.3758/s13415-015-0347-6) contains supplementary material, which is available to authorized users.

In neuroscience and psychology, behavior is thought to rely on contributions from at least two instrumental decision systems, one “goal-directed” and another “habitual” (Dickinson, 1985). The goal-directed system learns about the contingency between actions and outcomes and ensures that behavior is appropriate given our current desire for these outcomes. When we learn new complex tasks, such as how to drive, our goal-directed system is actively engaged as we plan each maneuver. With practice, however, components of this task require less effort, and we find ourselves, for instance, changing gears or dimming lights automatically, which allows us to allocate attention to more important things (e.g., pedestrians). This automaticity reflects the contribution of our habit system, which is thought to enable actions that have been rewarded many times in the past to be “stamped in,” such that they can be rendered automatic in the future. Although the ability to form habits is useful for routine tasks such as driving, too much reliance on habits can be problematic when responding to changing circumstances in which flexibility is required: for example, when we go abroad and must overcome the well-established habit of driving on a particular side of the street. More seriously, an accumulation of evidence has suggested that excessive habit learning is central to disorders of compulsivity, including obsessive-compulsive disorder (OCD; Gillan & Robbins, 2014) and substance dependence (Dickinson, Wood, & Smith, 2002; Sjoerds et al., 2013), which are characterized by cycles of maladaptive, repetitive behavior.

The existence of these two systems, and assays of their relative control over behavior in different circumstances or populations, are largely inferred from post-training outcome devaluation procedures (Adams & Dickinson, 1981), in which the value of a desirable outcome is reduced—for example, through selective satiety (Balleine & Dickinson, 1998; Tricomi, Balleine, & O’Doherty, 2009) or taste aversion (Adams & Dickinson, 1981). The ability to alter behavior when an outcome’s value changes demonstrates that a representation of the expected consequences of an action is guiding our choices, the hallmark of goal-directed control. Conversely, continued responding in spite of outcome devaluation is the defining feature of habits. Using outcome devaluation, it has been established that habits can become dominant following practice or “overtraining” (Adams, 1982; Tricomi et al., 2009), when the action–outcome contingency is lessened (Dickinson, Nicholas, & Adams, 1983), and also under conditions of stress (Schwabe & Wolf, 2009) and associative interference (de Wit, Niry, Wariyar, Aitken, & Dickinson, 2007). Moreover, the outcome devaluation methodology has been utilized to delineate neural loci associated with the motivational sensitivity of behavior in both humans and rodents (Balleine & O’Doherty, 2010). Although the caudate nucleus (or dorsomedial striatum, in rodents) and the ventromedial prefrontal cortex (vmPFC) promote outcome-sensitive action selection (de Wit, Corlett, Aitken, Dickinson, & Fletcher, 2009; de Wit et al., 2012; Valentin, Dickinson, & O’Doherty, 2007; Yin, Ostlund, Knowlton, & Balleine, 2005), the putamen (or dorsolateral striatum, in rodents) is thought to act in an opposing direction, supporting habit formation (Tricomi et al., 2009; Yin, Knowlton, & Balleine, 2004).

Although it is clear that the outcome devaluation technique has been extremely fruitful (having arguably laid the foundation for this entire field of study), it suffers from one key limitation: It cannot determine whether outcome-insensitive behaviors arise due to a strengthening of stimulus–response habits, a weakening of goal-directed choices, or a combination of both. More importantly, because this methodology examines only previously acquired behaviors, it does not speak to the learning mechanisms that give rise to either system’s tendencies, and in particular, it does not reveal how experiences during learning (such as the amount or the nature of training) ultimately produce a dominance of goal-directed or habitual actions. In the present study, we sought to connect goal-directed and habitual behaviors to learning mechanisms that have been hypothesized to produce them.

A popular computational framework posits that two update rules operate in parallel to learn action preferences during trial-and-error instrumental learning, known as “model-based” and “model-free” reinforcement learning (Daw, Niv, & Dayan, 2005; Keramati, Dezfouli, & Piray, 2011; Pezzulo, Rigoli, & Chersi, 2013). These theories formalize and generalize classical associative-learning conceptions of instrumental behavior, which envision goal-directed and habitual choices as arising from learned action–outcome or stimulus–response associations, respectively (Dickinson & Balleine, 1994). Suggesting a neural grounding for these behaviors, the model-free system is based on temporal difference learning theories (Sutton & Barto, 1998) of the dopaminergic midbrain, which suggest that one way in which actions acquire value is based on their history of reward, a process associated with phasic changes in the firing rate of dopamine neurons in the midbrain (Schultz, Dayan, & Montague, 1997). In contrast, model-based computations achieve greater accuracy and flexibility by evaluating candidate actions or sequences of actions on the basis of a learned cognitive map (the eponymous “model”) of their predicted outcomes (Tolman, 1948), and combining these predictions with separately learned information about the current incentive values of those outcomes (Doya, 1999; Gläscher, Daw, Dayan, & O’Doherty, 2010). The contributions of these two learning rules to trial-by-trial preference adjustments are distinguishable when learning multistep decision tasks, and the hallmarks of both rules have been reported in both human (Daw, Gershman, Seymour, Dayan, & Dolan, 2011) and rodent (Akam, Dayan, & Costa, 2013; Miller, Erlich, Kopec, Botvinick, & Brody, 2014) behavior. The relative contributions of these mechanisms to learning vary according to individual differences (Daw et al., 2011), aging (Eppinger, Walter, Heekeren, & Li, 2013), and psychiatric disorders (Voon et al., 2014). Moreover, individual differences in the propensity for model-based (over model-free) choice are positively correlated with gray matter volume in the vmPFC and caudate (Voon et al., 2014), regions that are critical for goal-directed control over action, in particular.

It has been proposed, though so far largely on theoretical grounds, that these two frameworks coincide: Specifically, that model-free and model-based learning (respectively) give rise to habits and goal-directed actions, as operationalized by devaluation sensitivity. This is because model-free learning is based entirely on one’s prior experience with reward, and therefore cannot use new information about the outcome value (including devaluation) to guide choices. Conversely, model-based selection should be associated with greater goal-directed sensitivity to devaluation, due to the online incorporation of outcome value information into choices between candidate actions (Daw et al., 2005; Dolan & Dayan, 2013). Thus, we set out to test whether these two constructs are indeed related, which (if it were true) would have implications for the learning processes that produce or protect against habits.

In Experiment 1, we tested these hypotheses in 90 participants recruited online using Amazon’s Mechanical Turk (AMT). The participants first completed two simultaneous two-step reinforcement-learning tasks that previously have been used to dissociate the contributions of model-based and model-free learning to choice behavior (Daw et al., 2011; Otto, Gershman, Markman, & Daw, 2013; Otto, Raio, Chiang, Phelps, & Daw, 2013). The tasks were structurally equivalent, but they were each associated with distinct rewards. Subsequently, we devalued one of these rewards (but not the other) and measured whether participants’ instrumental behavior would update in light of this change in the outcome value. Specifically, we assessed the extent to which participants would withhold responding toward devalued rewards, but continue to respond in order to gain still-valuable rewards. We then tested whether individual differences in the signatures of model-based and/or model-free learning during sequential decision-making were predictive of the devaluation sensitivity of the associations thereby acquired, revealing trial-by-trial learning mechanisms that contribute to, and/or protect against, forming habits. To verify our results, in Experiment 2 we carried out a conceptual replication study with 95 new participants. Here, we probed whether the learned preference for one action over another (within a single two-step choice task) was sensitive to devaluation, and tested whether this sensitivity was related to model-based and model-free learning, as in Experiment 1. This allowed us to explicitly test whether sensitivity to devaluation relates to the manner in which associations are learned through experience.

## Experiment 1

### Method

#### Participants

This study was conducted online using Amazon’s Mechanical Turk (AMT). Recruitment and exclusion was conducted on a rolling basis until a target sample size of 90 participants had been reached. It was necessary to recruit 111 participants to meet this target. As has been suggested for behavioral experiments on AMT, we defined the following a priori exclusion criteria to ensure data quality (Crump, McDonnell, & Gureckis, 2013; Simcox & Fiez, 2014). Participants were excluded (but still paid) if they missed more than 10 % of the trials (*n* = 18), had implausibly fast reaction times (i.e., ±2 *SD*s from the mean; *n* = 2), or responded with the same key on more than 90 % of trials (*n* = 1). The participants were recruited from the USA only, had been paid for 95 % of their previous work on AMT, and were 18 years of age or older. In all, 42 males and 48 females were included in the study, with ages ranging from 18 to 63 (*M* = 33.15 years, *SD* = 10.96). They were paid $2 for participation, in addition to a bonus payment that reflected the proportion of coins earned during training (*M* = $0.53, *SD* = 0.04). Participants could not take part in the experiment more than once or restart the experiment.

In order to obtain reliable learning data from AMT, a recent study suggested that it is necessary that participants first pass a comprehension test (Crump et al., 2013). This ensures that all those taking part have read and understood the instructions. Our participants needed 100 % accuracy on a seven-item comprehension test (provided in the supplementary materials) in order to take part in the present study. If any item was answered incorrectly, participants were sent back to the beginning of the instructions. They could not progress to the main test or become eligible for payment unless they passed this test. There was no constraint on the number of times that participants could repeat the instructions prior to taking part.

#### Reinforcement learning task

**Fig. 1:**
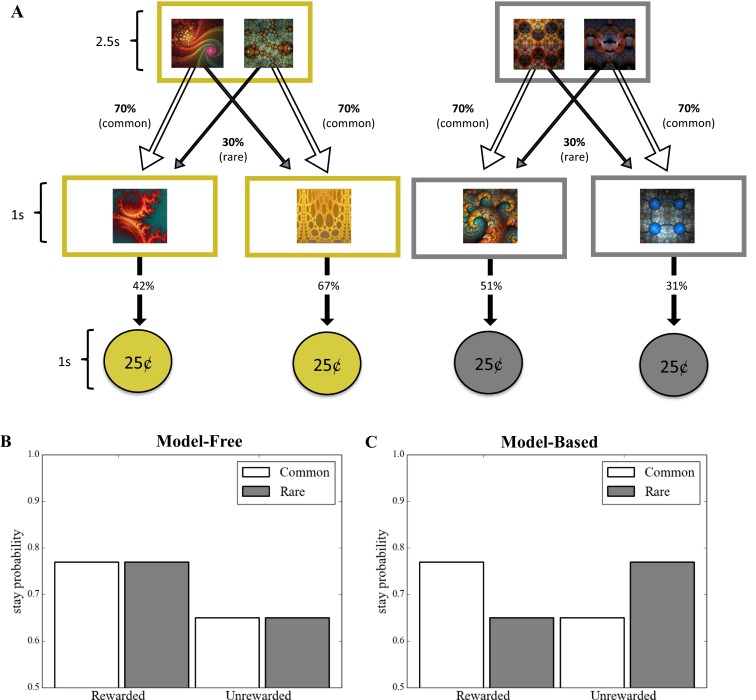
Experiment 1: Reinforcement-learning task. Participants entered one of two start states on each trial, which were associated with the receipt of gold and silver coins, each worth 25¢. Participants had 2.5 seconds (s) to make a choice, costing 1¢, which would commonly (70 %) lead them to a certain second state and rarely lead them to the alternative second state (30 %). No choices were made to the second state; each second state has a unique probability of reward that slowly changed over the course of the experiment. (B) Graph depicting a purely model-free learner, whose behavior is solely predicted by reinforcement history. (C) A purely model-based learner’s behavior, in contrast, is predicted by an interaction between reward and transition, such that behavior would mirror the model-free learner only when the transition from the initial choice to the outcome was common. Following rare transitions, a purely model-free learner would show the reverse pattern

On each trial, participants were presented with a choice between two fractals, each of which commonly (70 %; see Fig. 1A, white arrows) led to a particular second state displaying another fractal. These second-state fractals were termed “coin-boxes,” since they each had some probability (between .25 and .75) of being rewarded with a coin worth 25¢. On 30 % of trials (“rare” transition trials; Fig. 1A, gray arrows), choices uncharacteristically led to the alternative second state. A purely model-free learner would make choices irrespective of the transition structure of the task (i.e., whether a transition was rare or common), and would only show sensitivity to whether or not the last action had been rewarded (Fig. 1B). A model-based strategy, in contrast, is characterized by sensitivity to both prior reward and the transition structure of the task. For example, take the case in which a choice is followed by a rare transition to a second state, and that second state is rewarded. In this situation, a model-based learner would tend to switch choices on the next turn, because this would be more likely to return the learner to that second state (Fig. 1C). A model-free learner would make no such adjustment based on the transition type.

Before starting the task, participants completed a training session, which comprised written instructions (provided in full in the supplementary materials), the viewing of 20 trials demonstrating the probabilistic association between the second-stage fractals and coin rewards, and completion of 20 trials of active practice with the probabilistic transition structure of the task. The number of times that participants failed the comprehension test that followed these instructions was used as a covariate in our subsequent analyses, accounting for general comprehension ability.

To permit comparisons of behavior in relation to devalued and nondevalued rewards (described later), participants played two interleaved two-step Markov decision process (MDP) games, for gold and silver coins, respectively. At the start of each trial, participants entered either of two possible conditions (games), gold or silver. These were entirely independent of one another and were discriminable by the choices available (fractal images), the color of the border on the screen (gold or silver), and the type of coin available in that condition (Fig. 1A). Participants were instructed that any coins they earned during the task would be stored in a container of the corresponding color and converted to money at the end of the experiment. However, they were also informed that these containers stored a finite number of coins and that once a container became full, they would not be able to keep any more coins of that color.

Participants had 2.5 s in which to make a response using the left (“E”) and right (“I”) keys following presentation of the first-state choice. If no response was made within the time window, the words “no response” were presented in the center of the screen in red letters, and the next trial started. It cost 1¢ (0.01 USD) to make a choice on each trial, and “–1¢” was presented in red letters at the top right of the screen after each choice was made, to denote the cost incurred. If a choice was made, the selected fractal moved to the top center of the screen and shrunk in size. A new, second-state fractal appeared in the center of the screen and was followed by a coin or a zero, indicating that the participant had not been rewarded on that trial. The probability that each second-stage fractal would be followed by a coin changed slowly over trials (independent Gaussian random walks, *SD* = 0.025, with reflecting boundary conditions at 25 % and 75 %). Note that similar tasks used previously had contained an additional choice between two more options at each second-stage state, which was eliminated here for simplicity. Since the effect of model-based learning is mediated by the state identity—rare or common—this did not affect the logic of the task or its analysis.

#### Devaluation procedure

**Fig. 2:**
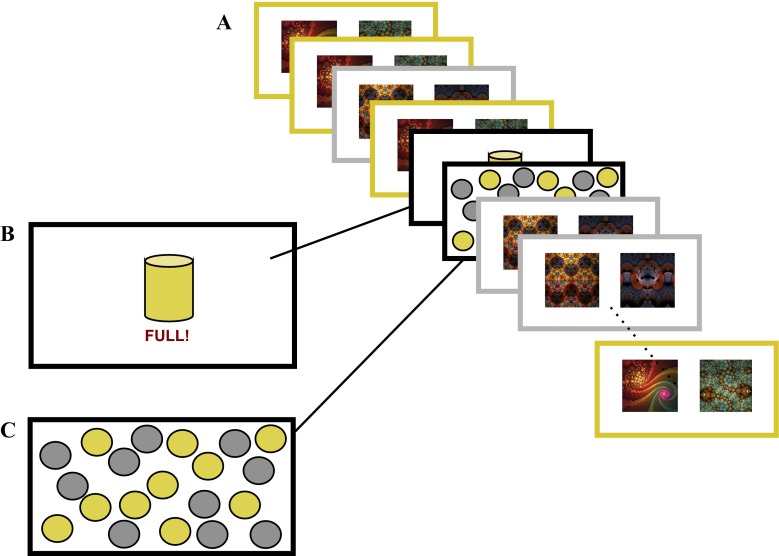
Experiment 1: Devaluation and consumption tests. (A) The 24-trial devaluation stage consisted of presentations of the first-stage choices only; that is, participants did not transition to the second stages and never learned the outcomes of their choices. This ensured that responding during the devaluation test was dependent only on prior learning. They were informed that the task would continue as before, but that they would no longer be shown the results of their choices. (B) After four trials of experience with the concealed trial outcomes, one type of coin was devalued by informing participants that the corresponding container was completely full. (C) This trial was followed by a consumption test, in which participants had 4 s to freely collect coins using their mouse. Next they completed the 20 test trials, in which habits were quantified as the difference between the numbers of responses made to the valued and devalued states

Once 200 trials of the sequential decision-making task had been completed, participants were informed that one of the containers became full, devaluing that coin type such that collecting these coins could no longer add money to their take-home bonus (Fig. 2B). Since it cost 1¢ (0.01 USD) to make a choice on each trial of the game, when a coin becomes devalued, an individual who behaves in a goal-directed manner should withhold responding in the condition associated with the devalued coins in order to avoid the unnecessary loss of 1¢ per trial. In contrast, if the habit system has gained control over action, an individual should continue to respond in both valued *and* devalued conditions at a cost of 1¢ per trial.

To exclude the possibility that new learning contributed to devaluation test performance, outcomes were not shown to participants during the test stage (Fig. 2A; de wit et al., 2009; Tricomi et al., 2009). Participants were warned about this change in task procedure, told that they would no longer see the results of their choices (i.e., whether or not they got a coin), but apart from that, nothing about the game had changed. We gave participants four trials with no feedback prior to devaluing one of the coins. This was done in order to allow participants the opportunity to learn about the change in feedback delivery prior to devaluation (and so not to conflate the two). Participants were alerted to this change in procedure: They were informed that the task would continue as before, but that they would no longer be shown the results of their choices. Following these trials, a screen indicated to participants that one of their containers (counterbalanced across participants) was completely full (Fig. 2B). A total of 20 post-reinforcer devaluation trials were presented in this test—ten trials per state, presented in a random order.

#### Consumption test

Outcome devaluation studies in rodents and humans have typically used primary reinforcers such as food to test the efficacy of devaluation by testing actual consumption of the item. Since the devaluation manipulation used in the present study was symbolic, we carried out an analogous consumption test to quantify the extent to which the devaluation manipulation was effective in reducing the incentive value of the devalued coin. Following the devaluation procedure, in which participants were shown that one of their containers was full, they were instructed that they would be given 4 s to collect as many coins as they pleased from a display of gold and silver coins (ten each; Fig. 2C) by clicking with their mouse. If devaluation was effective, participants should collect more of the valued than of the devalued coins.

During the main task—specifically, at Trials 105 and 135—participants received warnings that the to-be-devalued and then the to-remain-valued container, respectively, were half-full. These warning trials were followed by consumption tests. This served to familiarize participants with the finite storage capacity of the containers and consumption test procedure prior to devaluation.

#### Data analysis

Logistic regression analyses were conducted using mixed-effects models implemented with the lme4 package in the R programming language, version 3.0.2 (http://cran.us.r-project.org). The model tested for the effects of reward (coded as *rewarded* 1, *unrewarded* –1) and transition type (coded as *common* 1, *rare* –1) on the preceding trial in predicting each trial’s choice (coded as *switch* 0 and *stay* 1, relative to the previous choice). States were treated independently (i.e., had distinct stimuli and reward probabilities), and as such were treated independently in the analysis. For example, for a given trial in which participants made a choice in the gold state, the reward and transition variables in the model pertained to the previous trial experienced in the gold state, not necessarily the last trial experienced by the participant (which might have been in the silver state). In other words, if a participant made a choice in the gold state, we were interested in the extent to which their prior experience in that gold state had influenced the current choice. Within-subjects factors (the intercept, the main effects of reward and transition, and their interaction) were taken as random effects—that is, they were allowed to vary across participants. Critically, to test the hypothesis that devaluation sensitivity is associated with model-based learning during training, we included devaluation sensitivity (*z*-scored) as a between-subjects predictor and tested for interactions with all other factors in the model. We quantified devaluation sensitivity as the difference between the numbers of responses in the valued and devalued states. We hypothesized that we would find a significant three-way interaction between reward, transition, and devaluation, such that greater sensitivity to devaluation would be predictive of greater model-based control over action (the results are in Table 1 below). In the syntax of the lme4 package, the specification for the regression was Stay ~ Reward * Transition * Devaluation + (1 + Reward * Transition | Subject).

**Table 1:** Experiment 1: Results of logistic regression predicting stay probability

Coefficient	*β* (*SE*)	*z* Value	*p* Value
**(Intercept)**	**1.05 (0.09)**	**11.47**	**<.0001** ^***^
**Reward**	**0.68 (0.04)**	**17.69**	**<.0001** ^***^
Transition	–0.01 (0.02)	–0.56	.574
Devaluation	0.06 (0.09)	0.61	.541
**Reward × Transition**	**0.08 (0.03)**	**2.32**	**.020** ^*^
Reward × Devaluation	–0.003 (0.04)	–0.07	.945
Transition × Devaluation	0.01 (0.02)	0.47	.638
**Reward × Transition × Devaluation**	**0.10 (0.03)**	**3.02**	**.003** ^**^

Significant effects are bold. Reward (*rewarded* 1, *unrewarded* –1) and transition type (*common* 1, *rare* –1) are random effects predictors (within subjects), and devaluation sensitivity (*z*-scored) during the habit test is a fixed effect (between subjects). A significant main effect of reward and a significant Reward × Transition interaction indicate that, in line with previous studies, participants used a mixture of model-free and model-based learning, respectively. Importantly, a significant interaction between reward, transition, and devaluation reveals that individuals who behaved habitually at test were less likely to have used a model-based learning strategy during instrumental learning. ^*^ *p* < .05, ^**^ *p* < .01, ^***^ *p* < .001

Additionally, we carried out a second analysis aimed at corroborating the relationship between learning and devaluation sensitivity, but using devaluation sensitivity as the dependent variable, a specification that would more naturally reflect our causal hypothesis. For this, individual betas from a more basic model (i.e., like the one described above, but omitting devaluation sensitivity as a between-subjects predictor) were first extracted. Individual betas for the Reward × Transition interaction were termed the “model-based index,” and individual betas for reward were termed the “model-free index.” These indices (betas) were used as predictors in a linear model with devaluation sensitivity as the dependent variable. This analysis was therefore similar to the first analysis, although it was less sensitive because it failed to account for uncertainty in estimating the per-subject model-based indices. Nonetheless, it allowed us to test a causal assumption of our hypotheses, in so far as we can speak of causality in regression, that the reinforcement-learning dynamics during training would be predictive of sensitivity to devaluation of these same associations (the results are in Table 2). Alternative linear models were tested, with devaluation sensitivity as the dependent variable, including general task comprehension (the number of times that participants failed the instruction comprehension test) and consumption sensitivity as predictors. This allowed us to assess whether the relationship between devaluation and the model-based index could be explained by failures in either of these two aspects of task comprehension.

**Table 2:** Experiment 1: Results of linear model predicting devaluation sensitivity

Predictor	*β* (*SE*)	*t* Value	*p* Value
**(Intercept)**	**3.42 (0.42)**	**8.084**	**<.001** ^***^
Model-free index	0.18 (0.43)	0.428	.669
**Model-based index**	**1.26 (0.43)**	**2.950**	**.004** ^**^

Significant effects are bold. The “model-based index” (standardized individual Reward × Transition interaction betas) predicted individual sensitivity to devaluation, *p* = .004, whereas the “model-free index” (standardized individual reward betas) did not. ^**^ *p* < .01, ^***^ *p* < .001

Correlational analyses were conducted using Spearman’s rho. Finally, we complemented our main regression analysis with a full computational reinforcement-learning model, for which the methods are detailed in the supplementary materials.

### Results

We examined participants’ trial-by-trial adjustments in choice preferences during the initial learning task. Consistent with previous studies using similar learning tasks (Daw et al., 2011; Daw et al., 2005; Otto, Gershman, et al., 2013; Otto, Raio, et al., 2013), we found that they used a mixture of model-based and model-free learning strategies, evidenced by the presence of both a main effect of reward (*p* < .0001; the hallmark of model-free learning) and a Reward × Transition interaction (*p* = .02; i.e., model-based learning—see Table 1 and Fig. S1).

**Fig. 3:**
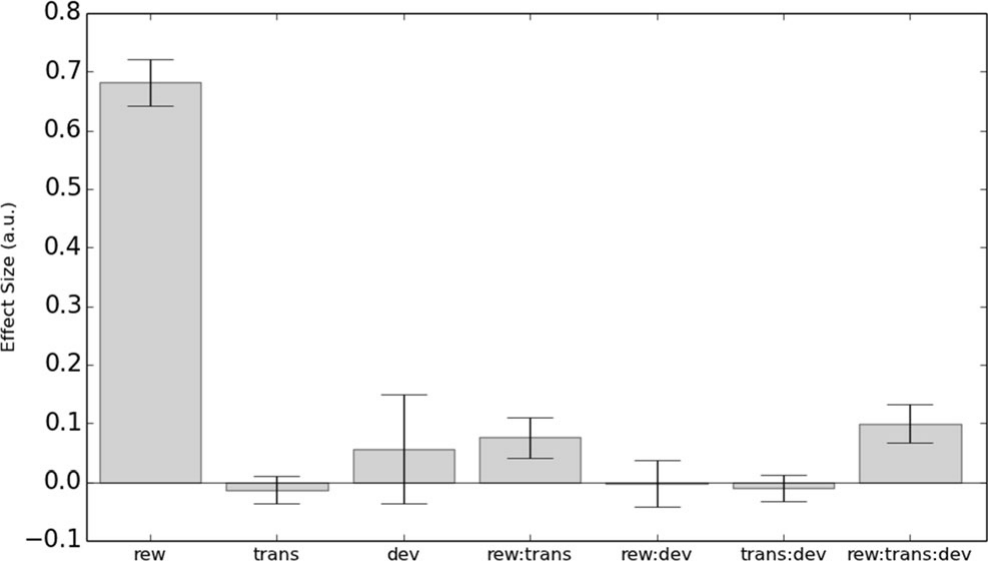
Experiment 1: Effect sizes (beta weights) from the logistic regression model (Table 1). Significant effects were observed for reward (model-free, *p* < .001), the Reward × Transition interaction (model-based, *p* = .020), and the predicted three-way interaction of reward, transition, and devaluation sensitivity (*p* = .003). rew = reward, trans = transition, dev = devaluation sensitivity

To assess whether model-based or model-free learning strategies were associated with the formation of devaluation-insensitive habits, we tested for the presence of (1) Reward × Devaluation and (2) Reward × Transition × Devaluation interactions. These significant interactions would indicate that a relationship existed between habits and the strengths of model-free and model-based learning, respectively. We found evidence in support of the latter hypothesis, such that participants who were more model-based during training also showed larger goal-directed sensitivity to devaluation in the habit test, (*β* = 0.1, standard error [*SE*] = 0.03, *p* = .003; see Table 1 and Fig. 3). No such relationship was seen for model-free responding. Since devaluation sensitivity scores were standardized for inclusion in the regression model, the estimated coefficients in Table 1 imply that an increase of one standard deviation in devaluation sensitivity doubles the observable effect of model-based learning, whereas if devaluation sensitivity is one standard deviation below the mean, it eliminates model-based learning altogether.

**Fig. 4:**
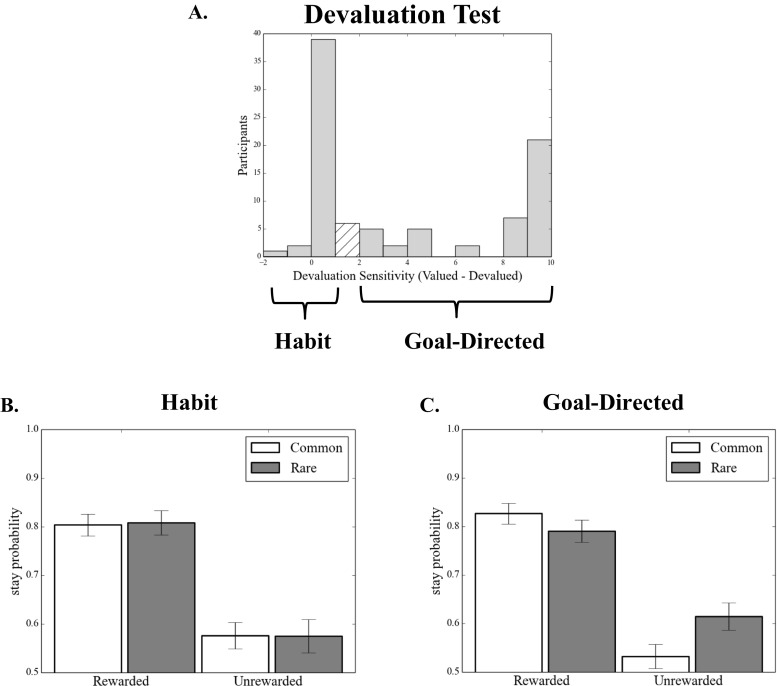
Experiment 1: Model-based learning and habit formation. (A) Histogram displaying devaluation sensitivity in the entire sample in Experiment 1. *Devaluation sensitivity* is defined as the difference between the numbers of valued and devalued responses performed in the test stage, with larger numbers indicating greater sensitivity to devaluation. To illustrate the relationship between model-based learning and habit formation, a median split divides the sample into (B) habit (devaluation sensitivity < 1) and (C) goal-directed (devaluation sensitivity > 1) groups. Those who displayed habits at test showed a marked absence of the signature of model-based learning, *p* < .003

We confirmed the relationship between a tendency toward model-based learning and the subsequent devaluation sensitivity of the acquired behaviors in a second version of the analysis, in which the devaluation sensitivity was taken as the dependent variable and indices of model-based and model-free learning from the training phase were used to predict it. Accordingly, across participants, the individual Reward × Transition interaction betas (“model-based index”) estimated from the basic learning model (i.e., with no between-subjects predictors included) significantly predicted devaluation sensitivity (*β* = 1.26, *SE* = 0.43, *p* = .004), whereas the reward betas (“model-free index”) did not (*β* = 0.18, *SE* = 0.43, *p* = .669) (Table 2). The distribution of devaluation sensitivity scores was bimodal, with peaks at 0 and 10 (Fig. 4A). A score of 10 indicated *maximum devaluation* (goal-directed; all possible responses made in the valued state and no responses made in the devalued state), whereas a score of 0 indicated that a participant *responded equally frequently in both states*, indicating that his or her behavior did not change selectively for the devalued coin (habit). Therefore, we illustrate this effect using a median split (Figs. 4B and C), which shows that participants who remained goal-directed in the devaluation test (i.e., “goal-directed”) showed the characteristic mixture of both model-based and model-free learning during training, whereas those who formed habits (“habit”) showed the complete absence of a model-based instrumental learning strategy (Fig. 1B).

#### Reinforcement-learning model

The aforementioned regression analyses considered only events taking place on the trial immediately preceding choice and were originally motivated as a simplified limiting case of a more elaborate computational model of how these two strategies learn action preferences progressively over many trials (Daw et al., 2011). To verify that the relationship between model-based learning and subsequent devaluation sensitivity would remain when we fully considered incremental learning taking place over many trials, we additionally fit a computational model to the choice data, in which separate model-based and model-free systems contributed to individual participants’ behavior (Daw et al., 2011). The model-free system uses temporal-difference learning to incrementally update action values on the basis of their history of reward. The model-based system, in contrast, constructs models of both the transition and reward structures of the task and integrates this information in order to prospectively assign values to possible actions (see the supplemental materials for a detailed description of the computational model).

We estimated the free parameters of this model for each individual participant, and also their group-level distributions, by fitting them to the observed sequences of choices and rewards (using Markov chain Monte Carlo sampling over a hierarchical Bayesian model). Notably, the relative balance between model-based and model-free learning is, in this framework, captured by a single weight parameter (*w*), which is larger when model-based learning is relatively more dominant. At the group level, we estimated a regression slope relating devaluation sensitivity, across subjects, to *w*, and found that this was significantly positive, such that the result mirrored those in the regression analysis [median = 0.86, 95 % confidence interval: lower tail 0.04, upper tail 1.94]. Greater sensitivity to devaluation was associated with a greater relative contribution of model-based than of model-free learning signals to choice. This relationship was specific to *w*, in that no significant relationship was observed between devaluation sensitivity and the additional parameters in our model, including learning rate and perseveration.

#### Consumption test

We verified the efficacy of the devaluation procedure by using a post-devaluation consumption test. We found a main effect of coin value on consumption, *F*(1, 89) = 247.28, *p* < .0001, so that, as predicted, participants collected more valued (*M* = 5.41, *SD* = 1.96) than devalued (*M* = 0.6, *SD* = 1.33) coins. This confirmed that the devaluation manipulation was effective. Individual differences in consumption sensitivity, like devaluation sensitivity, were quantified as the difference between the consumption of valued and devalued coins, in which a score of 10 indicated a *maximal shift in incentive value toward valued coins*, and 0 reflected *no differentiation between valued and devalued coins*. There was no significant correlation between devaluation sensitivity and consumption sensitivity (Spearman’s *r* = .12, *p* = .278), indicating that continued responding in the devaluation test was indicative of habit—that is, was unrelated to the current incentive value of the outcomes of actions. We furthermore tested whether consumption explained away the relationship between model-based-index and devaluation sensitivity, by including it as an additional explanatory variable in our linear regression in which per-subject devaluation sensitivity was the dependent measure and the per-subject model-based index, consumption, and their interaction were predictors. Consumption did not predict devaluation (*p* = .23), nor did it interact with the model-based index (*p* = .631) or explain away the relationship between the model-based index and devaluation (which remained significant at *p* = .0341).

#### General comprehension

Finally, we tested whether a more general measure of comprehension ability, the number of times that participants failed the instruction comprehension test, was associated with model-based performance. The number of fails was marginally associated with the model-based index (Spearman’s *r* = –.2, *p* = .063), such that better general comprehension ability (fewer fails) was associated with a greater model-based index (note that this was not replicated in Exp. 2 below). Importantly, when we repeated the regression analysis above, but replacing consumption with comprehension, we found that comprehension did not predict devaluation performance (*p* = .792), nor did it interact with the model-based index (*p* = .739) or explain away the relationship between the model-based index and devaluation (*p* = .039).

## Experiment 2

In Experiment 1, we operationalized habits by the decision whether or not to engage in *any* response for the devalued option, mimicking the classic definition from free-operant responding in rodents (Adams, 1982). However, because the devaluation probe (the decision to engage or not to engage) was distinct from the choice between fractals (i.e., *which* response to make), a question may arise whether the devaluation probe depended on the particular associations learned experientially during the task, or whether it could instead depend upon more general information that was acquired from the task instructions. Formally, the expected value of responding following devaluation was the product of the current value of the coin times the chance that any subsequently chosen response within the MDP would produce that coin, which implied that the decision whether to respond at all should ultimately depend on the same (model-based or model-free) learned associations that drove the choice between responses. However, in Experiment 1, it might be possible in principle to short-circuit this computation and conclude that responding for the devalued outcome was worthless, because participants were instructed that the color of the border presented on the screen (gold or silver) indicated the possible outcome associated with a response in each MDP. Thus, the result from Experiment 1 could speak to a more general relationship between a tendency to be model-based and a tendency to be goal-directed on separate “tasks”, rather than to the more specific hypothesis that associations learned in model-based fashion are more sensitive to subsequent devaluation. To address this possibility, in Experiment 2 we tested a separate sample of 95 participants with a modified version of the paradigm that could more directly probe the sensitivity to devaluation of fractal choice associations, which participants acquired in the training phase using either model-based or model-free strategies (or a mixture of the two).

### Method

**Fig. 5:**
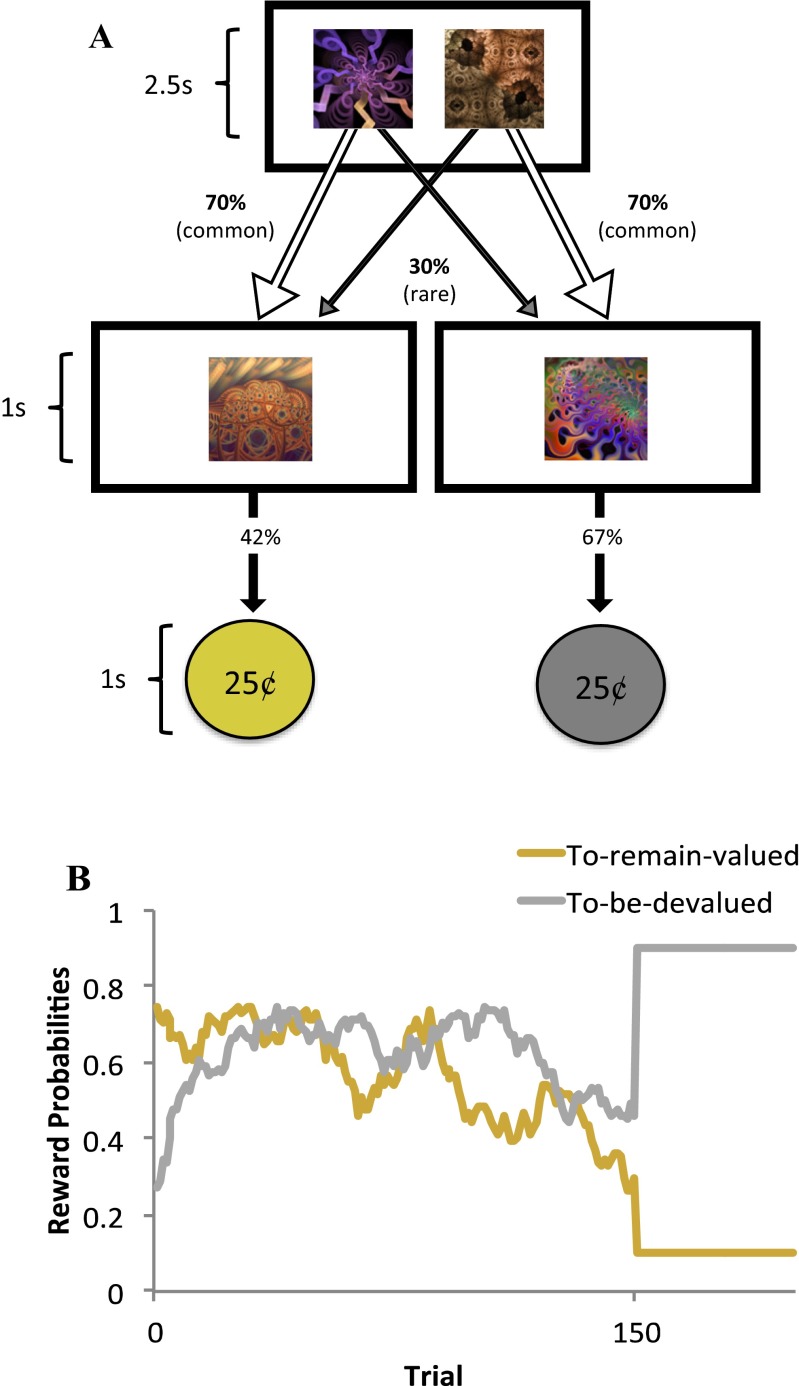
Experiment 2: Reinforcement-learning task. (A) Participants entered the same starting state on each trial and had 2.5 s to make a choice between two fractal stimuli that always appeared in this state. One fractal commonly (70 %) led to one of the second-stage states and rarely (30 %) led to the other. In contrast to Experiment 1, each second-stage state was uniquely associated with a certain type of coin (gold or silver). (B) For the first 150 trials, reward probabilities (the chance of winning a coin in a given second-stage state) drifted slowly over time according to Gaussian random walks. For the next 50 trials, the reward probabilities stabilized at .9 and .1, for the second-stage states associated with the to-be-devalued and to-remain-valued outcomes, respectively. This served to systematically bias all participants toward making the action that would later be devalued. Devaluation was randomized across coin colors and reward drifts

In all, 39 males and 56 females were included in Experiment 2, and their ages ranged from 19 to 70 (*M* = 34.33 years, *SD* = 12.03). In this version of the task, we used a single MDP (instead of the two used in Exp. 1). The two second-stage states were associated with gold and silver coins, respectively (Fig. 5). There was no option to withhold responses, which did not come at a cost. The outcomes were devalued in exactly the same way as in Experiment 1, but prior to the outcome devaluation, we stabilized the reward probabilities at .9 and .1 for the second-stage states associated with the to-be-devalued and to-remain-valued coin colors, respectively. The second-stage state that produced the to-be-devalued coin type was always stabilized to .9, serving to bias participants to this choice prior to devaluation, so that we could examine subsequent habitual responses toward the devalued outcome against a high pre-devaluation baseline. Devaluation was randomized across coin colors and reward drifts. The main MDP task comprised 150 trials, which were followed by 50 trials of stable reward probabilities (only the former were used to assess trial-by-trial learning mechanisms). As in Experiment 1, we included four trials with no feedback prior to devaluing one of the outcomes, in order to decorrelate these occurrences. The habit test comprised ten trials with no feedback after participants had been alerted that one of their coin containers was full. To test for sensitivity to devaluation, we measured the proportion of valued responses selected in the test stage—that is, trials on which participants did not choose the devalued outcome. This measure of devaluation sensitivity was then entered into a mixed-effects logistic regression with the effects of reward, transition, and the intercept taken as random effects, and devaluation sensitivity as a fixed effect (as per the main model in Exp. 1). Ninety-nine participants were tested in Experiment 2, of which three were excluded because they missed more than 10 % of the total trials during the main MDP task, and one was excluded for having an implausibly fast reaction time (>2 *SD*s lower than the mean).

### Results

During the two-stage decision task, participants again showed evidence of both model-free learning (a main effect of reward) and model-based learning (an interaction between reward and transition, both at *p* < .0001; see Table 3). In a stable phase at the end of this task, we successfully induced a choice preference for the action associated with the to-be-devalued coin color by giving it a high probability of reward for 50 trials. A one-sample *t* test confirmed that the proportion of trials on which participants chose the action associated with the high reward probability (*M* = .716, *SD* = .27) was greater than chance (.5), *t*(94) = 7.71, *p* < .001. The proportion of trials on which the high-probability action was selected was not correlated with the model-based index (Spearman’s *r* = .114, *p* = .272) or the model-free index (Spearman’s *r* = .129, *p* = .212).

**Table 3:** Experiment 2: Results of logistic regression predicting stay probability

Coefficient	*β* (*SE*)	*z* Value	*p* Value
**(Intercept)**	**1.62 (0.12)**	**14.10**	**<.0001** ^***^
**Reward**	**1.06 (0.08)**	**12.52**	**<.0001** ^***^
Transition	0.05 (0.03)	1.55	.121
**Devaluation**	**0.26 (0.11)**	**2.25**	**.024** ^*^
**Reward × Transition**	**0.24 (0.04)**	**5.67**	**<.0001** ^***^
Reward × Devaluation	–0.04 (0.08)	–0.49	.625
Transition × Devaluation	0.01 (0.03)	0.32	.751
**Reward × Transition × Devaluation**	**0.15 (0.04)**	**3.55**	**<.001** ^**^

Significant effects are bold. Reward (*rewarded* 1, *unrewarded* –1) and transition type (*common* 1, *rare* –1) are random effects predictors (within subjects), and devaluation sensitivity (*z*-scored) during the habit test is a fixed effect (between subjects). Here, *devaluation sensitivity* refers to the proportion of presses toward still-valuable outcomes in the habit test. As in Experiment 1 (see Table 1), we observed a significant main effect of reward, a significant Reward × Transition interaction, and a significant three-way interaction between reward, transition, and devaluation, revealing that individuals who behaved habitually at test (i.e. did not preference valued over devalued choices) were less likely to have used a model-based learning strategy during instrumental learning. ^*^ *p* < .05, ^**^ *p* < .01, ^***^ *p* < .001

**Fig. 6:**
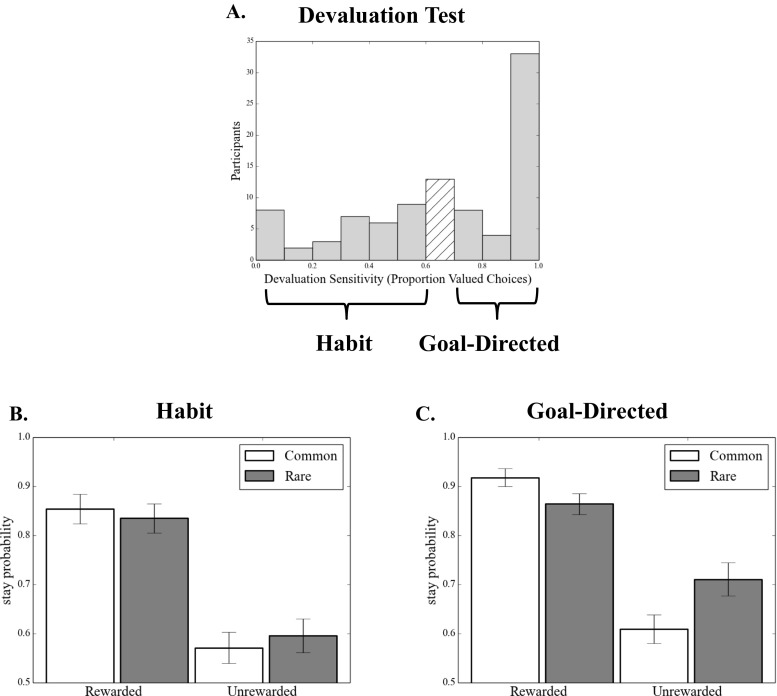
Experiment 2: Model-based learning and habit formation. (A) Histogram displaying devaluation sensitivity in the entire sample from Experiment 2. Here, *devaluation sensitivity* is defined as the proportion of valued choices (over total choices) made at the test stage, with larger numbers indicating greater sensitivity to devaluation. To illustrate the relationship between model-based learning and habit formation, a median split divides the sample into (B) habit (devaluation sensitivity < .6) and (C) goal-directed (devaluation sensitivity > .6) groups. Consistent with Experiment 1, the participants who displayed habits in Experiment 2 (i.e. failed to prefer valued over devalued choices) showed a reduction in the signature of model-based learning, *p* < .001

In the devaluation test, in line with the results of Experiment 1, we again observed a range of devaluation sensitivities across the population, ranging between exclusive choice of the devalued (habit) and still-valued actions (Fig. 6A). Critically, when entering devaluation sensitivity as an explanatory factor in the analysis of the two-stage decision task, we observed a significant three-way interaction between reward, transition, and devaluation sensitivity (Table 3). In line with the results from Experiment 1, this indicated that greater sensitivity to devaluation was associated with an increase in model-based learning (Figs. 6B and C). This confirmed that when behavior is acquired in a model-based manner, that behavior is subsequently more sensitive to outcome devaluation—that is, less likely to be habitual. Additionally, we observed a main effect of devaluation sensitivity on stay/switch behavior, such that better sensitivity to devaluation was associated with a greater tendency to repeat the same action on subsequent trials, irrespective of reward and transition. These results were fully echoed in fits using the full computational model, which are presented in the supplement (Table S2).

As in Experiment 1, we also recapitulated this analysis using a fixed-effects model in which devaluation sensitivity was the dependent measure. Again, we found that the model-based index predicted devaluation (*p* = .0002), whereas the model-free index did not (*p* = .70). We again tested whether this relationship might be explained by some more general factor, and found that this was not the case. The results from these analysis are presented in the supplement. Unlike in Experiment 1, we found that consumption was related to devaluation sensitivity (*p* = .05), but importantly, as in Experiment 1, its inclusion in the model did not explain away the relationship between model-based learning and devaluation sensitivity (*p* = .006). Likewise, task comprehension was related to devaluation sensitivity here (*p* = .018), but again this did not explain away the relationship between model-based learning and habits (*p* = .002). Finally, unlike in Experiment 1, task comprehension did not significantly correlate with the model-based index (Spearman’s *r* = –.16, *p* = .13).

## Discussion

In two complementary experiments, we combined an outcome devaluation probe with a multistage decision task in order to characterize the learning dynamics that give rise to actions or habits. In Experiment 1, habits were operationalized traditionally—that is, as failures to withhold costly responding in light of devaluation (Adams, 1982). In Experiment 2, we extended this result to habits defined by a preference for a devalued choice over a still-valued choice, which have been utilized more recently in human studies (Schwabe & Wolf, 2009). The latter experiment also ensured that any effects of devaluation were mediated by associations learned experientially during the task. Across both experiments, we found that the extent to which actions were learned using model-based updating during instrumental training predicted their later sensitivity to devaluation. Put another way, individuals who did not show the signatures of model-based learning were more likely to display habits when the outcomes associated with these learned behaviors were devalued. These data suggest that signatures of model-based instrumental learning are an appropriate formalization of goal-directed behavior, and importantly, that these signatures (which predict the ultimate formation of goal-directed actions vs. habits) are detectable during initial, trial-by-trial acquisition. Interestingly, we did not find evidence for a separate relationship between a signature of model-free learning and devaluation sensitivity.

Our results converge with recent data showing a correlation, across a small sample of participants, between the results from two separate tasks, one probing sequential decision-making and another assessing sensitivity to devaluation (Friedel et al., 2014). By instead testing within a single task the devaluation sensitivity of the same associations whose model-based learning was assessed, through our present design we were able to address the hypothesized computational mechanism by which model-based learning produces devaluation-sensitive behaviors. The present study also controlled for the efficacy of the devaluation manipulation at reducing the desirability of outcomes using consumption and general task comprehension, factors that might contribute to either or both tasks and confound their relationship. The Friedel et al. study is complementary to the present one in effecting devaluation by using a specific satiety manipulation (Valentin et al., 2007) that is more similar to the procedures used in typical rodent studies than is our more explicit, cognitive manipulation.

More generally, the direct relationship between learning type and devaluation sensitivity that we demonstrated is consistent with recent patterns of findings using either procedure separately. For instance, disorders of compulsivity, which are associated with deficits in goal-directed devaluation sensitivity (Dickinson et al., 2002; Gillan et al., 2014; Gillan et al., 2011; Sjoerds et al., 2013), are also accompanied by impairments in model-based, but not model-free, learning (Voon et al., 2014). Likewise, stress responses are associated with deficient model-based, but not model-free, learning (Otto, Raio, et al., 2013), converging with earlier work showing that stress impairs devaluation sensitivity in both humans (Schwabe & Wolf, 2009) and rodents (Dias-Ferreira et al., 2009). All of these results suggest that evoking a model-based strategy during learning—that is, incorporating the relationship between candidate actions and future outcomes and the current incentive values of outcomes into the decision process—is protective against habit formation. These results are also consistent with the apparent overlap in the neural loci associated with devaluation sensitivity and model-based learning (Balleine & O’Doherty, 2010). As we outlined in the introduction, these processes share a dependence on the caudate nucleus and the vmPFC (Daw et al., 2011; de Wit et al., 2009; de Wit et al., 2012; Gillan et al., 2015; Valentin et al., 2007; Voon et al., 2014; Yin et al., 2005).

Although (the absence of) model-based learning predicted the subsequent dominance of habits, the level of model-free learning did not. Negative results must be interpreted with caution, but this may be consistent with an overall structure of behavioral control in which habits represent a more robust, prepotent default, which can be effortfully overridden by model-based decisions if these have been learned (Otto, Skatova, Madlon-Kay, & Daw, 2015). That we found no evidence for a relationship between individual differences in model-free learning and habit formation may also be consistent with neuroimaging data, since there have as yet been fewer clear indications that habits and model-free learning coincide neurally than that goal-directed action and model-based learning do (see Daw & O’Doherty, 2014, for a review). Therefore, one important implication of the present (null) result is that model-free learning may be entirely unrelated to habit-forming tendencies (Dezfouli & Balleine, 2013). Another possibility is that, rather than two independent dimensions, the balance between model-based and model-free learning might be better understood as a single parameter along a spectrum (as with *w* in our computational model), which (in this task and analysis) may be more sensitively detected by the model-based index. Consistent with either view, the model-based index has repeatedly proved to be more sensitive to manipulations and individual differences (Otto, Gershman, et al., 2013; Otto, Raio, et al., 2013; Otto et al., 2015; Voon et al., 2014; Wunderlich, Smittenaar, & Dolan, 2012). Finally, we cannot exclude the possibility that the influence of individual differences in model-free learning on habit formation is tied to external promoters of habit, such as the duration of training experienced (Tricomi et al., 2009) or the extents to which learning is implicit or explicit (the former of which would be expected to promote model-free learning; Frank, Rudy, Levy, & O’Reilly, 2005). Future studies should aim to tackle these issues and determine whether a relationship indeed exists between incremental reinforcement learning and habit formation (which is not explained by goal-directed learning).

It is notable that model-based performance was found to depend partly on task comprehension. The extent to which this is particularly relevant to this task (and not a common feature of most experimental paradigms) is an open question, but nonetheless, comprehension should be handled carefully, particularly when using this task in challenged populations. In Experiment 1, the effect of model-based learning observed was smaller than the effects observed in Experiment 2 and in prior studies using the original version of the sequential task. This is not surprising, given that participants were required to learn two independent transition structures in Experiment 1, rather than just one. This change likely rendered model-based learning more difficult. Additional differences between Experiment 1 and the original version of this paradigm (Daw et al., 2011) included the added cost of responding, the absence of second-stage choices, and a lower overall payment amount (a feature of both Experiments 1 and 2). Whereas a previous study has shown that varying the rate of remuneration between levels that has been typical with AMT and in-person experiments has little effect on behavior in a category-learning task (Crump et al., 2013), the first two changes described above may have affected task behavior—for instance, by encouraging participants to deliberate more over their choices or making value representations less distinct between states, respectively. In future work, it will be possible to test these possibilities by manipulating the various task features separately, but the important point for the present study is simply that, relative to previous variants of the task, we retained the key ability to detect significant contributions of model-based and model-free learning to choice, and thus to test their relationships to devaluation.

In addition to suggesting that model-based learning signals are an appropriate formalization of goal-directed control over action, the data from the present study provide support for the attractive possibility that biases toward habit formation can be identified during trial-by-trial learning, allowing researchers to avoid the practical challenges associated with using one-shot devaluation tests (Seger & Spiering, 2011). For instance, a trial-by-trial rather than a one-shot probe approach is much more amenable to methodologies such as unit recording or functional magnetic resonance imaging, for which averaging over many trials is typically necessary. Similarly, and crucially, the outcome devaluation test can speak only indirectly to the dynamic mechanisms supporting these respective systems. Reinforcement-learning frameworks offer the computational specificity that one-shot assay tests lack in this regard, and thus have already been used to identify more distributed neural signatures of instrumental choice (Daw et al., 2011) that may be critical for advancing our understanding of the relationship between brain function and compulsivity in psychiatric disorders. Overall, this framework provides renewed scope to directly investigate the hitherto opaque learning mechanisms through which training duration, contingency, and value interact to produce habits in both healthy animals and clinical populations.

## Electronic supplementary material

Below is the link to the electronic supplementary material.

ESM 1 (PDF 382 kb)
