# Supplementary material for: Model-based learning protects against forming habits
Source: Cogn Affect Behav Neurosci. 2015 Mar 24;15(3):523–36. doi: 10.3758/s13415-015-0347-6 (PMC4526597; doi:10.3758/s13415-015-0347-6)
Supplement: Supplementary file 1 — (PDF 382 kb) [file 13415_2015_347_MOESM1_ESM.pdf]

Supplementary Materials

Model-based learning protects against forming habits

*Claire M Gillan[123], A Ross Otto[4], Elizabeth A Phelps[145] and Nathaniel D Daw[14]

Affiliations

[1]Department of Psychology, New York University, 6 Washington Place, New York, NY 10003, USA
[2]Department of Psychology, University of Cambridge, Downing Site, Cambridge CB2 3EB, United Kingdom
[3]Behavioural and Clinical Neuroscience Institute, University of Cambridge, Downing Site, Cambridge CB2 3EB, United Kingdom
[4]Center for Neural Science, New York University, 4 Washington Place, New York, NY 10003, USA
[5]Nathan Kline Institute, Orangeburg, NY 10962, USA

Corresponding Author
*Claire M Gillan
Department of Psychology, New York University, 6 Washington Place, New York, NY 10003, USA.
Tel. +1 347 630 5137
claire.gillan@gmail.com

Acknowledgements
This work was funded by a Sir Henry Wellcome Postdoctoral Fellowship (101521/Z/12/Z) awarded to CM Gillan. The authors report no conflicts of interest.

Reinforcement Learning (RL) Model

The reinforcement-learning (RL) model used in this study is based on a hybrid of model-free ($Q(\lambda)$) and model-based (explicit computation of top level state-action values), as utilized in previous studies (Daw, Gershman, Seymour, Dayan, & Dolan, 2011; Otto, Raio, Chiang, Phelps, & Daw, 2013). Experiment 1 comprised two conditions (gold and silver), each of which had three unique states (first stage: s_A; second stage: s_B and s_C). The first stage in each condition had two available actions (a_A and a_B), while the second stages did not, but rather were probabilistically followed by reward (where probabilities drifted gradually over time). Note that the implementation of the computational model for Experiment 2 was exactly the same as for Experiment 1, except that only one state was in play, rather than two. The hybrid model employed consists of model-based and model-free subcomponents, both of which estimate a state-action value function, $Q_{MF}(s_A, a)$ (model-free) and $Q_{MB}(s_A, a)$ (model-based), which maps each possible action to its expected future reward. On trial t, we denote the first-stage state (always s_A) by $s_{1,t}$, the second-stage states by $s_{2,t}$, the chosen first-stage action by a_t, and the second-stage rewards as r_t .

<u>*Model-free component*</u>

For the model-free algorithm, we used Q(λ), temporal difference (TD) learning (Peng & Williams, 1996), which updates the value for the visited state-action pair at $s_{1,t}$ according to:

$$Q_{MF}(s_{1,t}, a_t) = Q_{MF}(s_{1,t}, a_t) + \alpha\delta_{1,t}$$

where $\delta_{1,t}$ is the reward prediction error (RPE) at state 1, trial t:

$$\delta_{1,t} = Q_{MF}(s_{2,t}) - Q_{MF}(s_{1,t}, a_t)$$

and α is a learning rate parameter, which is constant across conditions and states for a given individual. Thus, for the first-stage choice, the RPE is driven based on the second-stage value, $Q_{MF}(S_{2,t})$. Second-stage values are themselves updated according to:

$$Q_{MF}(s_{2,t}) = Q_{MF}(s_{2,t}) + \alpha\delta_{2,t}$$

where the RPE is determined by whether or not the trial was rewarded, r_t:

$$\delta_{2,t} = r_t - Q_{MF}(s_{2,t})$$

We treat the gold and silver states as separate from one another; that is, Q-values for the

gold and silver states were learned independently of one another, as was the structure of the task. The model uses an eligibility trace to propagate second-stage reward information to the first-stage values. Specifically, at the end of each trial, the first-stage values are updated according to:

$$Q_{MF}(s_{i,t}, a_t) = Q_{MF}(s_{i,t}, a_t) + \alpha\lambda\delta_{2,t}$$

where λ is an eligibility trace decay parameter (Sutton & Barto, 1998). We assume that eligibility traces are reset to 0 between episodes (i.e., that eligibility does not carry over from trial to trial). Additionally, at the end of each trial, we decayed the Q values for all of the non-selected actions by multiplying them by $1 - \alpha$ (Ito & Doya, 2009; Lau & Glimcher, 2005). This decay makes the present model correspond more closely to the one-trial-back regression model described in the main text, in the limit as $\alpha \rightarrow 1$.

<u>*Model-based component*</u>

In general, a model-based RL algorithm works by learning a transition function (mapping state-action pairs to a probability distribution over the subsequent state), and immediate reward values for each state, then computing cumulative state-action values by iterative expectation over these. Specialized to the structure of the current task, this amounts to, first deciding which first-stage action maps to which second-stage state (because subjects were instructed that this was the structure of the transition contingencies), and second, learning reward values for each of the second-stage states.

We modeled transition learning by assuming subjects simply chose between the two possibilities: $P(s_B|s_A, a_A) = 0.7, P(s_C|s_A, a_B) = 0.7$ or vice versa $P(s_B|s_A, a_A) = 0.3, P(s_C|s_A, a_B) = 0.3$, with $P(s_B|s_A, a_B) = 1 - P(s_B|s_A, a_A)$ and $P(s_C|s_A, a_B) = 1 - P(s_C|s_A, a_A)$, according to whether more transitions had thus far occurred to s_B following a_A plus s_C following a_B, or vice versa to s_C following a_A plus s_B following a_B.

At the second stage (where immediate rewards were offered), the problem of learning immediate rewards is equivalent to that for TD above, because $Q_{MF}(s_{2t})$ is just an estimate of the immediate reward r_t; with no further stages to anticipate, and the SARSA learning rule reduces to a delta rule for predicting the immediate reward. Thus, the two approaches coincide at the second stage, and we define $Q_{MB} = Q_{MF}$ at those states. Critically, the top level model-based values are defined from both the transition and reward estimates using the Bellman Equation (Bellman, 1957):

$$Q_{MB}\left(s_A, a_{A_j}\right) = P(s_B|s_A, a_j)\, Q_{MF}(s_B) + P(s_C|s_A, a_j)\, Q_{MF}(s_C)$$

where we have assumed these are recomputed on each trial from the current estimates of the transition probabilities and rewards.

Choice rule

Finally, to connect the values to choices, we use a softmax choice rule, which assigns a probability to each action according to the weighted combination of the two state-action value estimates $Q_{net}(s,a) = wQ_{MB}(s,a) + (1-w)Q_{MF}(s,a)$. Q_{MB} and Q_{MF} , weighted according to a free parameter *w.* Choice is then softmax in the net state-action values.

The probability of each choice at the first stage is calculated, accordingly, as

$$P\left(a_t = a|s_{1,t}\right) = \frac{\exp[\beta \cdot\ Q_{net}\left(s_{1,t}, a\right)p \cdot rep(a)]}{\Sigma_{a'} \exp[\beta \cdot\ Q_{net}\left(s_{1,t}, a'\right)p \cdot rep(a')]}$$

where the inverse temperature parameter β governs the stochasticity of choices. The indicator function rep(a) is defined as 1 if a is the same one as was chosen on the previous trial, zero otherwise. Together with the "stickiness" parameter p, this captures first-order perseveration ($p > 0$) or switching ($p < 0$) in the first- stage choices (Lau & Glimcher, 2005).

Group-Level Modeling

Thus far we have described the modeling of a single subject's data. This model was embedded within a multi-level random effects model of the population variation in its parameters to estimate it for all subjects simultaneously. All of the free parameters of the model (α, λ, β, w , p) were taken as random effects, instantiated separately for each subject *s* from a common group level distribution. For all parameters, the group level distributions were Gaussian with free group-level mean (μ) and SD (σ), plus an additional free slope (e.g. β_d) allowing the parameter to scale, across subjects, with their (z-scored) devaluation score d_s. This was implemented identically in Experiments 1 and 2, using their respective indices of devaluation sensitivity. For instance:

$$\beta_s \sim Normal(\mu_\beta\ + \beta_d \cdot d_s, \sigma_\beta)$$

and analogously for p. However, for the parameters with support confined to [0,1] (α, λ, and *w*) the resulting variables were transformed according to a logistic sigmoid, e.g.:

$$w'_s \sim Normal(\mu_w + w_d \cdot d_s, \sigma_w)$$

$$w_s = \frac{1}{1 + \exp(-w_s')}$$

and similarly for the others.

We estimated the parameters of the group level distributions ($\mu_p, \sigma_p, p_d, \mu_\beta, \sigma_\beta, \beta_d, \mu_\alpha, \sigma_\alpha, \alpha_d, \mu_\lambda, \sigma_\lambda, \lambda_d, \mu_w, \sigma_w, w_d$) using uninformative priors: for the parameters with infinite support (β, p), the prior means and devaluation slopes were the broad *Normal*(0,10), the prior SDs, the heavy-tailed *Cauchy*(0,2.5). For the parameters with finite support (α, λ, and *w*), we selected narrower prior distributions so that the sigmoid-transformed parameters were roughly uniform in [0,1] a priori; prior means for these parameters were *Normal*(0,2.5); prior variances *Cauchy*(0,2) and prior devaluation slopes *Cauchy*(0,2.5).

Estimation

We estimated the joint distribution of the parameters of the model, conditional on all subjects' observed choices and rewards. For this, we used Markov Chain Monte Carlo (MCMC) techniques (specifically the No-U-Turn variant of Hamiltonian Monte Carlo) as implemented in the Stan modeling language (v2.5, 2014). Given a probabilistic generative model (the above equations) and a subset of observed variables, MCMC techniques provide samples from the conditional joint distribution over the remaining random variables. We ran four chains of 4,000 samples each, discarding the first 2,000 samples of each chain for burn-in. We examined the chains visually for convergence and also computed Gelman and Rubin's (1992) potential scale reduction factors. For this, large values indicate convergence problems, whereas values near 1 are consistent with convergence. We ensured that these diagnostics were less than 1.2 for all variables.

Supplementary Regression Analyses

Given our relatively large sample size and the presence of some unique task features, we carried out additional exploratory analyses that may be of general interest (but were unrelated to our hypotheses). In each experiment, there was no significant relationship between age (Exp1: β=0.013, SE=.022, p=.554; Exp2: β=0.007, SE=.026, p=.802) or gender (coded Female=1, Male=-1) (Exp1: β=-0.024, SE=.022, p=.266; Exp2: β=0.019, SE=.027, p=.475) and model-based learning. Nor was there a significant relationship between age (Exp1: β=0.034, SE=.026, p=.198; Exp2: β=0.102, SE=.07, p=.128) or gender (Exp1: β=-0.038 SE=.026, p=.148; Exp2: β=0.068, SE=.07, p=.316) and model-free learning. In Experiment 1, unlike Experiment 2 and the original version (Daw et al., 2011), there were two independent states, one gold and another silver. Therefore, on some trials the previous state experienced was different from the current state. We tested

if this affected choice behavior by including state information as an additional predictor in our logistic regression analysis of the learning task behavior (same state:1, different state: -1, relative to the previous trial). This factor was included as a random effect (i.e., allowed to vary across subjects) and interacted with the other explanatory variables. We found that when the State on the current trial was the same as that on the previous one, participants were more likely to make the same choice (main effect of State β =0.13, SE=.033, $p<.001$), were more model-free (interaction of State by Reward, β =0.26, SE=.03, $p<.001$) and trended towards being more model-based (three-way interaction of State, Reward, and Transition, β =-.048, SE=.03, $p=.068$). Therefore, not unexpectedly, the three basic effects on the choices were more robustly observed when participants made choices in the same state as the immediately previous trial, whereas these effects were diminished when experience with the other state intervened.

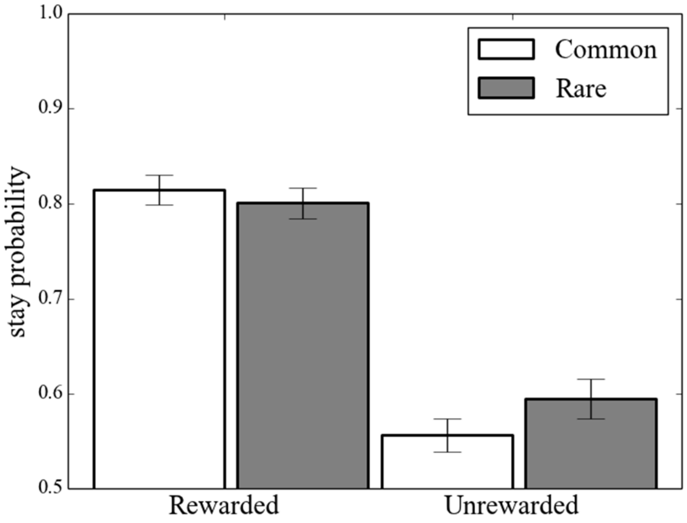

Figure S1. Result from the main logistic regression model collapsed across all subjects in Experiment 1

As reported in the main text, we observed that subjects used a mixture of model-based and model-free learning strategies, evidenced by the presence of both a main effect of reward (*p*<.0001; model-free) and a reward x transition interaction (*p*=.02; model-based).

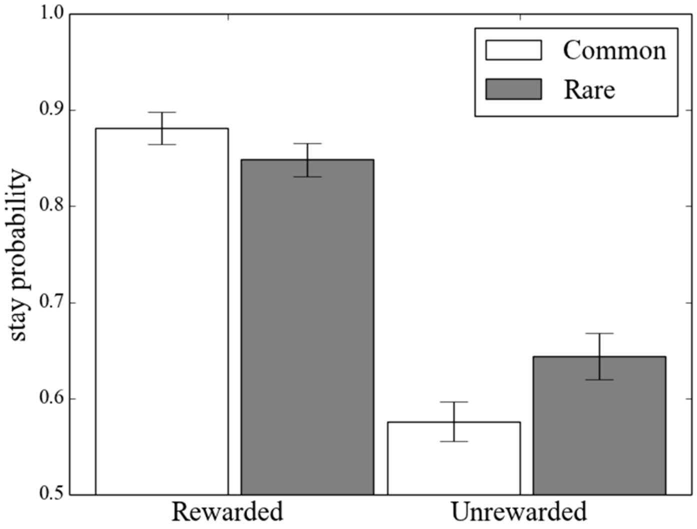

Figure S2. Result from the main logistic regression model collapsed across all subjects in Experiment 2
As in Experiment 1, we observed that subjects used a mixture of model-based and model-free learning strategies, evidenced by the presence of both a main effect of reward (*p*<.0001; model-free) and a reward x transition interaction (*p*<.0001; model-based).

Table S1. Experiment 1: Group-level estimates of the effect of each free parameter on devaluation performance in the computational model.

	Parameter effects on devaluation				
	αDev	$pDev$	λDev	βDev	$wDev$
**Upper 95%**	0.40	0.03	3.01	0.74	**2.20**
**Median**	0.11	-0.08	0.07	0.34	**1.01**
**Lower 95%**	-0.17	-0.19	-2.21	-0.06	**0.07**

For each parameter, the median posterior estimate is given, together with the 95% confidence intervals. Only the slope of *wDev* is significantly different from zero.

Table S2. Experiment 2: Group-level estimates of the effect of each free parameter on devaluation performance in the computational model.

	Parameter effects on devaluation				
	αDev	$pDev$	λDev	βDev	$wDev$
**Upper 95%**	0.3	**0.37**	1.46	0.87	**4.97**
**Median**	-0.12	**0.21**	-0.76	0.35	**2.92**
**Lower 95%**	-0.54	**0.04**	-3.29	-0.16	**1.44**

For each parameter, the median posterior estimate is given, together with the 95% confidence intervals. The slope of *wDev* and *pDev* (i.e. perseveration) are significantly different from zero.

Task Instructions for Experiment 1

SCREEN 1

Welcome. Today you will be playing a game where you can earn some extra money.

On every turn, you will make a choice between two boxes, which will bring you to another box, which may or may not contain a coin.

At the end of the game, we will convert a **proportion** of what you have won into real money, which you can keep in addition to your hit payment. Simply put, the more coins you collect in the game, the more cash we will pay you when you are finished.

Coins can be **gold** or **silver**, and are always worth 25c, regardless of color. The border of the screen on each turn will let you know whether gold or silver coins are available.

You must read all of the instructions carefully. There will be a quiz before you begin the game and if you do not answer all of the questions correctly, you will be sent back to the beginning and will need to re-read them.

SCREEN 2

Before you start making choices, we will have a tutorial to show you how the game works. There are two things for you to learn in order to do well. We will practice these separately.

The first is:

You need to keep track of which boxes have the highest chance of having a coin inside. If a box contains a coin, it will appear below the box. If not, you will see a zero.

Every time you find a box, the computer will decide whether or not to give you a coin based on a 'chance', which has been assigned to that box. Some boxes have a higher chance of having a coin inside than others. Importantly, the chances of each box containing a coin will change slowly, and independently, over time. It is your job to keep track of which boxes are **currently** better than others and to try and get to these boxes.

There are no strange patterns to this game, such as a box containing coins on every other choice. The computer is not trying to play tricks on you; it strictly works on the chance assigned to each box, which will change slowly over time.

SCREEN 3

We will now let you practice this part of the game. You need to track which 'coin boxes' have a better chance of having coins than others.

Two of the coin boxes that we will show you sometimes contain silver coins, but will **never** contain gold coins. Likewise, the other two boxes will sometimes contain gold coins, but will **never** contain silver coins.

Unlike the real game, you won't be making any choices in this tutorial. Instead you simply need to pay attention to what happens on-screen so that you can work out which coin boxes are better than others. Learning how to do this will help you win money later on in the game.

SCREEN 4 (following 20 practice trials passively learning which boxes are more likely to yield coins)

Good. Hopefully, you saw that some boxes had a higher chance of having a coin inside than others. Also you probably noticed that even 'good' coin boxes didn't have a coin every single time.

The second thing you need to learn is how to make choices that will bring you to coin boxes that are currently good.

At the start of each turn, you will be able to choose between two boxes, which appear on the left and right. One of these boxes usually brings you to **one** of the coin boxes, and the other box usually brings you to the **other** coin box. For example, one box that you choose might bring you to one of the coin boxes on 7 out of 10 turns. But that means that on 3 out of 10 turns, it will take you to another box, by mistake. These chances are fixed, so you just need to learn these rules once. This is unlike the goodness of the coin boxes, which will change slowly over time during the game.

To sum-up: learning which boxes are more (or less) likely to bring you to each coin box is very important to playing the game well. If you can do this, you will be able to make good choices that will bring you to the coin boxes that are currently best.

We will practice making the choices now. We won't show you the coins in this tutorial, so that you can concentrate on learning which choices bring you to which coin boxes.

Press the:

E key to choose the LEFT BOX

I key to choose the RIGHT BOX

You have a limited amount of time to make each choice. Try practicing a few times right now.

SCREEN 5 (following 20 practice trials actively learning the transition structure through making choices and viewing their consequences)

Good. Hopefully you learned which choices usually lead to which coin boxes. You also will have noticed that by 'usually', we mean 70% of the time. That means that 30% of the time, you ended up in the other coin box.

It is important to know that the chance that a certain choice takes you to a certain coin box does not change over time. This means that if one box **usually** takes you to a certain coin box, that relationship will stay the same throughout the game.

SCREEN 6

Now that you understand the two parts you have practiced, we will remind you how they fit together in the game you are about to play. On each turn, you have a choice between two boxes, you will choose a box that will take you to a coin box and you will see if it contains a coin. After you find out whether or not your box contains coins, you will go back to the start and make another choice and try to earn another coin and so on.

While some of the coin boxes may become very good at times (that is, they often contain a coin), these same coin boxes may become bad later in the game. **You need to stay on top of which coin boxes are best.** You must use this information to make good choices that are likely to bring you to the boxes that are currently good.

It costs you **1 cent** to choose between the boxes and play the game on each turn. We will deduct this from your bonus at the end of the game. This is a good deal, because if you know that a box is good, you will often find a 25 cent coin inside. If you choose not to open one of the boxes, or are too slow to respond, there is no way that you can get a 25 cent coin on that trial, but will save yourself 1 cent. Sometimes it might make sense to save yourself that 1 cent if you are really sure that there is nothing of value inside the coin boxes.

SCREEN 7

IMPORTANT: We will store your gold and silver coins in separate containers, one for gold coins and one for silver coins, so we can keep track of how much you have won. Please note that if either of these containers fills up completely during the game, you have maxed-out on storing that kind of coin! You won't be able to keep any more coins of that color for the rest of the game. That means even if you continue to find that kind of coin inside boxes, **we won't store them for you**, and you won't get to keep them. We will alert you if at any point your containers get half-way full, and again if they get completely full.

SCREEN 8 (Comprehension Test)
(correct answers are emboldened)

Here is a short quiz to test if you understood the instructions correctly.

If you missed any important things, we will bring you to go through the instructions again.

Does it cost money to play the game on each trial?

- -I don't know
- **-Yes, it costs 1 cent**
- -Yes, it costs 5 dollars
- -No, it is free to play

Does the goodness of a coin box change over time?

- -I don't know
- -No, some boxes are good and others are bad. This will not change
- -No, coins are delivered randomly and no boxes are ever good or bad
- **-Yes! It changes slowly over time**

Does the chance that a certain choice leads to a certain coin box change over time?

-I don't know

-No, the chances are about 70% and this will not change

-No, the relationship between chances and coin boxes is completely unpredictable

-Yes! It changes slowly over time

If the silver container fills up, what will happen?

-I don't know

-I can start keeping my extra silver coins in the gold container

-**I have collected all of the silver coins that I will be allowed to keep**

-I lose all of my silver coins

If the gold container fills up, what will happen?

-I don't know

-I can start keeping my extra gold coins in the silver container

-I have collected all of the gold coins that I will be allowed to keep

-I lose all of my gold coins

Are silver and gold coins worth the same amount of money?

-I don't know

-Silver coins are more valuable than gold coins

-Gold coins are more valuable than silver coins

-Yes! They are both worth 25c

Can some boxes contain both silver and gold coins at different times?

-I don't know

-No. Boxes contain either silver or gold coins, never both

-Sometimes boxes will have both types of coins inside

-Yes! Boxes often have both types of coins inside

SCREEN 9

Well done! You answered the questions correctly!

Before you start the game, **let's review**. Your aim is to collect as much money as possible by finding coins. To do this, you will make choices between pairs of boxes which will bring you to another box that may contain a coin. Often one box will be better than another because it pays you more often. However, the goodness of the coin boxes will

change slowly over time, so you need to stay on top of which coin boxes are currently good.

Getting to the best coin boxes relies on your choice between the two boxes at the start of each turn. You have already learned how some choices are more likely to take you to certain coin boxes. This will not change over the game.

You will need to put these parts together to do well at the game and win as much money as possible. You need to keep track of which are the best coin boxes as that changes slowly, and make good choices that you think are likely to bring you to them.

Now we're going to play the real game where you can win money, which we will pay you as a bonus, in addition to your regular hit payment.

Use the **'E'** and **'I'** keys to choose the left and right boxes.

Miscellaneous Instructions

Consumption test

Free Coin Collection!

For a brief time, you can collect coins by clicking on them using the mouse/trackpad. Once collected, coins will disappear from the screen and be placed in their containers, if they are not already full.

You will have 4 seconds to collect as many coins as you like. Get ready!

Withholding outcome information

We will now no longer show you the results of your choices (i.e. whether or not you get a coin on each trial). Apart from that, nothing about the game has changed, and you should continue playing as before.

Supplementary References

Bellman, R. (1957). *Dynamic Programming*. Princeton, NJ: Princeton University Press.

Daw, N. D., Gershman, S. J., Seymour, B., Dayan, P., & Dolan, R. J. (2011). Model-Based Influences on Humans' Choices and Striatal Prediction Errors. *Neuron, 69*(6).

Gelman, A., & Rubin, D. (1992). Inference from iterative simulation using multiple sequences. *Statistical Science, 7*(4), 457-472.

Ito, M., & Doya, K. (2009). Validation of decision-making models and analysis of decision variables in the rat basal ganglia. *J Neurosci, 29*(31), 9861-9874.

Lau, B., & Glimcher, P. W. (2005). Dynamic response-by-response models of matching behavior in rhesus monkeys. *J Exp Anal Behav, 84*(3), 555-579.

Otto, A. R., Raio, C. M., Chiang, A., Phelps, E. A., & Daw, N. D. (2013). Working-memory capacity protects model-based learning from stress. *Proc Natl Acad Sci U S A, 110*(52), 20941-20946.

Peng, J. and Williams, R. J. (1996). Incremental multi-step Q- learning. *Machine Learning, 22*, 283-290.

Stan. (2014). Stan: A C++ Library for Probability and Sampling, Version 2.5. Retrieved October 15, 2014

Sutton, R., & Barto, A. (1998). *Reinforcement Learning: an Introduction*. Cambridge, Massachusetts: MIT Press.
